# Damping Ratio Estimation of Heavily Damped Structures Using State-Space Modal Responses

**DOI:** 10.3390/s25175416

**Published:** 2025-09-02

**Authors:** Jungtae Noh, Jae-Seung Hwang, Maria Rosa Valluzzi

**Affiliations:** 1 Department of Architectural Engineering, Dankook University, Yongin 16890, Republic of Korea; jungtae.noh@dankook.ac.kr; 2 School of Architecture, Chonnam National University, Gwangju 61186, Republic of Korea; 3 Department of Cultural Heritage, University of Padova, P.za Capitaniato 7, 35139 Padova, Italy; mariarosa.valluzzi@unipd.it

**Keywords:** vibration control system, heavily damped structure, non-classical damping, state-space-based mode decomposition, modal parameter estimation

## Abstract

Vibration control systems are extensively utilized in structures to enhance their resilience against earthquakes and wind forces. However, structures with significant damping exhibit atypical damping behaviors, which impose constraints on the effectiveness of traditional modal analysis methods for discerning modal responses and estimating properties. To surmount this challenge, a novel State-Space-Based Modal Decomposition approach is proposed in this study. The State-Space-Based Modal Decomposition technique adeptly extracts modal responses and identifies modal attributes from acquired data of highly damped structures. The approach accurately calculates damping ratios and natural frequencies by scrutinizing the power spectrum within the deconstructed modal response. The validity of this method is confirmed through a numerical simulation with a three-degree-of-freedom system equipped with oil dampers and experimentation of a structure outfitted with a tuned mass damper system. The findings underscore that the transfer function of the modal response in state-space encompasses both displacement and velocity transfer functions. The results demonstrate that precise estimation of modal parameters can be accomplished by suitably evaluating the participation ratio of the two response components.

## 1. Introduction

In single-degree-of-freedom (SDOF) systems, estimation of dynamic parameters such as natural frequency and damping ratio is relatively straightforward when the structural response is dominated by a single frequency. In contrast, for multi-degree-of-freedom (MDOF) systems, the modal matrix enables transformation of physical coordinates into modal coordinates, allowing for separation into independent SDOF modal responses. This process facilitates the identification of dynamic characteristics using system identification techniques. A key advancement in this area has been the application of Blind Source Separation (BSS) methods, including Independent Component Analysis (ICA) and Second-Order Blind Source Separation (SOBSS), which exploit high-order and second-order statistical properties of signals, respectively [1,2,3,4].

However, traditional BSS methods often rely on the assumption of classical damping, which assumes proportionality to mass and stiffness matrices. In many modern structures equipped with advanced damping systems—such as oil dampers and tuned mass dampers—this assumption does not hold. In such cases, modal orthogonality is violated, and classical modal decomposition methods fail to separate coupled responses effectively. To address these limitations, state-space-based mode decomposition methods have been proposed [5,6,7], offering improved mode separation under non-classical damping conditions.

Recent advancements have extended these approaches in multiple directions. Ref. [8] proposed a frequency domain state-space-based framework to enhance mode decomposition in non-classically damped structures. Their subsequent work introduced Kalman filtering for mode extraction [9] and later extended this to identify modal properties and loading information from output-only measurements [10]. While these methods improved decomposition accuracy, the frequency characteristics of modal transfer functions—especially the composition of the numerator—were not explicitly examined.

Ref. [11] proposed a sparse component analysis-based method for modal identification of non-classically damped structures, enhancing separation robustness. Similarly, ref. [12] employed AI-driven BSS to enable rapid operational modal analysis, yet lacked interpretability in terms of transfer function structure. Efforts to enhance accuracy under real-world conditions have also been made. Ref. [13] introduced methods for blind modal identification under non-stationary excitations, while ref. [14] validated the effectiveness of state-space decomposition techniques in systems with closely spaced modes. Nonetheless, a consistent issue across these studies is the limited focus on the monochromaticity or polychromaticity of modal transfer functions, especially in the context of displacement-velocity state-space formulations.

The importance of this distinction lies in the fact that, while the denominator of modal transfer functions typically reflects a single resonance corresponding to one natural frequency and damping ratio—thus exhibiting monochromatic behavior—the numerator can involve contributions from various dynamic interactions, especially in non-classically damped systems. This results in the presence of multiple frequency components, or polychromaticity, in the modal response even when the mode is theoretically separated.

This study addresses this critical gap by investigating the spectral structure of state-space modal responses, with particular focus on the polychromatic traits—that is, the presence of multiple non-resonant frequency components—in the numerator of the transfer function. These traits arise because state-space modal responses are constructed from both displacement and velocity states, which introduces phase and amplitude interactions that broaden the spectral content beyond a single frequency.

## 2. State-Space-Based Mode Decomposition

### 2.1. Real Number Mode Decomposition

This section introduces the theoretical foundation of the real number mode decomposition method. The equations of motion for a multi-degree-of-freedom (MDOF) structure can be represented in a state-space form:

(1) $$M\ddot{x}+C\dot{x}+Kx=Ef$$

(2) $$\dot{z}=Az+Bf$$

$$z=\left(\begin{array}{c} x\\ \dot{x} \end{array}\right),\;A=\left[\begin{array}{cc} 0 & I\\ -M^{-1}K & -M^{-1}C \end{array}\right],\;\;B=\left[\begin{array}{c} 0\\ -M^{-1}E \end{array}\right]$$

where ***M***, ***C***, ***K***, and ***E*** denote the mass, damping, stiffness, and external load (f) location matrices, respectively. When the stiffness matrix C involves non-classical damping, mode decomposition cannot be accomplished in the physical space, referred to as M(mass)-C(damping)-K(Stiffness) space. By converting the equations into state-space representation and performing mode decomposition, a complex modal matrix (T), modal response (η), and state variable (z) can be obtained:

(3) AT=TΛ

(4) z=Tη

where A and Λ denote a system matrix and eigenvalue, respectively.

Complex matrix (***T***) below and modal response (η) are always conjugates, while the state variables should be real numbers.

(5) $$T=\left[\Phi +i\Psi ,\;\;\;\Phi -i\Psi \right]$$

(6) $$\eta =\left(\begin{array}{c} p\left(t\right)+iq\left(t\right)\\ p\left(t\right)-iq\left(t\right) \end{array}\right)$$

where Φ and Ψ denote the real and imaginary components of the complex mode matrix T, respectively.

For the sake of convenience, time *t* can be omitted when expanding, and the state variables can be calculated.

(7) $$\begin{array}{c} z=\left(\Phi +i\Psi \right)\left(p+iq\right)+\left(\Phi -i\Psi \right)\left(p-iq\right)\\ =\Phi \left(2p\right)-\Psi \left(2q\right)\\ =\left[2\Phi -2\Psi \right]\left(\begin{array}{c} p\\ q \end{array}\right) \end{array}$$

Therefore, the real number modal response can be found by using the demixing matrix W:

(8) $$\left(\begin{array}{c} p\\ q \end{array}\right)=W^{T}z,\;\;W^{T}\;=[2\Phi ,-2\Psi {]}^{-1}$$

This indicates that the demixing matrix and modal responses in real numbers exist in the state-space. McNeill (2008) demonstrated that mode separation in the state-space can be achieved using the analytic signals obtained from a measured physical response to establish the state variable responses and the Second-Order Blind Identification (SOBI) technique.

Another approach involves converting a complex mode matrix into a real number mode matrix using the canonical form. The relationship between the canonical form ($\Lambda_{m}$) and complex eigenvalue (Λ) is as follows:

(9) $$\Lambda_{m}T_{m}=T_{m}\Lambda$$

$$\Lambda_{m}=\left[\begin{array}{cc} 0 & I\\ -\Omega & -\Sigma \end{array}\right],\;\Omega =diag\left({\omega_{i}}^{2}\right),\Sigma =diag\left(2\xi_{i}\omega_{i}\right)$$

where $\omega_{i}$ and $\xi_{i}$ are the i-th mode angular velocity and damping ratio, respectively.

$$T_{m}=\left[\begin{array}{cccccccc} 1 & 1 & 0 & 0 & 0 & 0 & 0 & 0\\ 0 & 0 & 1 & 1 & 0 & 0 & 0 & 0\\ \vdots & \vdots & \vdots & \vdots & \ddots & \ddots & \vdots & \vdots \\ 0 & 0 & 0 & 0 & 0 & 0 & 1 & 1\\ \lambda_{1} & {\lambda_{1}}^{*} & 0 & 0 & 0 & 0 & 0 & 0\\ 0 & 0 & \lambda_{2} & {\lambda_{2}}^{*} & 0 & 0 & 0 & 0\\ \vdots & \vdots & \vdots & \vdots & \ddots & \ddots & \vdots & \vdots \\ 0 & 0 & 0 & 0 & 0 & 0 & \lambda_{n} & {\lambda_{n}}^{*} \end{array}\right],\;\;\lambda_{i}=-\xi_{i}\omega_{i}+i\sqrt{1-{\xi_{i}}^{2}}\omega_{i}$$

Using Equations (2) and (6), the state variables from the real number mode (***r***), converted from the system matrix A with the canonical form, are as follows [8]:

(10) $$AT_{p}=T_{p}\Lambda_{m},\;\;\;\;\;T_{p}=T\;{T_{m}}^{-1}$$

(11) $$z=T_{p}r$$

Therefore, the real number modal response ***r*** is as follows:

(12) $$r=W^{T}z,\;\;\;\;\;W^{T}={T_{p}}^{-1}$$

State-space-based mode decomposition (SSBMD) can be applied to Equation (12) to separate modes in the state-space, as demonstrated by [8]. SSBMD utilizes state variables and their derivatives retrieved from integrating measured acceleration responses.

### 2.2. Polychromatic Characteristics of the State-Space Mode

The state variable derived from analytic signals or integrated responses contains combined heterogeneous polychromatic signals. Consequently, the resulting modal response also exhibits polychromatic features, in contrast to the monochromatic characteristics observed in the modal response derived from homogeneous signals in the MCK domain. This section evaluates the polychromatic attributes of the state-space mode. Equation (3) is then utilized on Equation (1) to compute the complex mode, including the conjugate particular modes:

(13) $$\left(\begin{array}{c} \eta {˙}_{i}\\ {\eta {˙}_{i}}^{*} \end{array}\right)=\left[\begin{array}{cc} \lambda_{i} & 0\\ 0 & {\lambda_{i}}^{*} \end{array}\right]\;\left(\begin{array}{c} \eta_{i}\\ {\eta_{i}}^{*} \end{array}\right)+\left(\begin{array}{c} b_{i}\\ {b_{i}}^{*} \end{array}\right)f$$

For simplicity, subscripts are omitted, and the modal responses are Laplace transformed.

(14) $$\begin{array}{c} \eta \left(s\right)=\frac{b}{s+\lambda }f\left(s\right)\;\\ \eta^{*}\left(s\right)=\frac{b^{*}}{s+\lambda^{*}}f\left(s\right) \end{array}$$

Plugging Equation (13) into Equation (7), the state variable is given as

(15) $$z\left(s\right)=\Phi \frac{\left(b+b^{*}\right)s+b\lambda^{*}+b^{*}\lambda }{s^{2}+\left(\lambda +\lambda^{*}\right)s+\lambda \lambda^{*}}+i\Psi \frac{\left(b-b^{*}\right)s+b\lambda^{*}-b^{*}\lambda }{s^{2}+\left(\lambda +\lambda^{*}\right)s+\lambda \lambda^{*}}$$

Here, assuming that the damping matrix has classical damping, the terms in the complex mode matrix can be rewritten as

(16) $$\Phi =\left(\begin{array}{c} 0\\ \Phi_{2} \end{array}\right)$$

(17) $$\Psi =\left(\begin{array}{c} \Psi_{1}\\ 0 \end{array}\right)$$

Substituting Equations (16) and (17) into Equation (15), the state variable with monochromatic properties only can be obtained as follows:

(18) $$z\left(s\right)=\left(\begin{array}{c} \Psi_{1}\frac{k_{1}}{s^{2}+\left(\lambda +\lambda^{*}\right)s+\lambda \lambda^{*}}\\ \Phi_{2}\frac{k_{2}s}{s^{2}+\left(\lambda +\lambda^{*}\right)s+\lambda \lambda^{*}} \end{array}\right)$$

where $k_{1}$ and $k_{2}$ are constant. Equations (15) and (18) reveal a unique natural frequency and damping ratio within the transfer function’s denominator, ensuring monochromaticity. While the numerator retains monochromatic attributes for classical damping, non-classical damping introduces polychromatic features, incorporating both Laplace variable s and a constant term.

Erroneous estimation of dynamic properties can occur by assuming exclusive monochromatic properties in modal responses of non-classically damped systems within the state-space. Therefore, it is vital to consider the polychromatic attributes of the modes that are decomposed in the state-space.

### 2.3. Damping Ratio Estimation Model

After recovering the SDOF response, various identification techniques can estimate its dynamic properties. In this context, the Virtual Dynamic Shaker (VDS) method is applied for dynamic property identification [15,16]. The VDS, serving as a virtual SDOF system model, employs modal response as input and derives the virtual system’s response as output. By adjusting the natural frequency and damping ratio that define the VDS, the damping ratio can be estimated from the modal response. The VDS, utilizing the modal response as input, is expressed as follows:

(19) $${\ddot{u}}_{t}+2\xi_{t}\omega_{t}\dot{u_{t}}+{\omega_{t}}^{2}u_{t}=\eta$$

where $u_{t}$, $\xi_{t}$, and $\omega_{t}$ denote the displacement, damping ratio, and natural angular velocity of the VDS, respectively, and the displacement can be expressed in the frequency domain.

(20) $$u_{t}\left(s\right)=\frac{\eta \left(s\right)}{s^{2}+2\xi_{t}\omega_{t}s+{\omega_{t}}^{2}}$$

The input for the VDS is the modal response (η), comprising displacement, velocity, and acceleration components. Under the assumption of a consistent external load spectrum and the VDS having the same natural frequency as the structure, the variance of the VDS response and output can be analytically derived. The subsequent equations depict the modal response using displacement, velocity, and acceleration components.

(21) $$\begin{array}{c} E\left[{u_{t}}^{2}|\eta =disp.\right]=\frac{\pi S_{o}}{8}\frac{1}{{\omega_{t}}^{7}}\frac{1+4\xi_{t}\xi_{s}}{\left(\xi_{t}+\xi_{s}\right)\xi_{t}\xi_{s}}\\ E\left[{u_{t}}^{2}|\eta =vel.\right]=\frac{\pi S_{o}}{8}\frac{1}{{\omega_{t}}^{5}}\frac{1}{\left(\xi_{t}+\xi_{s}\right)\xi_{t}\xi_{s}}\\ E\left[{u_{t}}^{2}|\eta =acc.\right]=\frac{\pi S_{o}}{8}\frac{1}{{\omega_{t}}^{3}}\frac{1}{\left(\xi_{t}+\xi_{s}\right)\xi_{t}\xi_{s}} \end{array}$$

Using the random damping ratio of the VDS, *a* and *b*, the ratio in Equation (22) indicates the displacement modal response as follows:

(22) $$R=\frac{b}{a}\frac{b+\xi_{s}}{a+\xi_{s}}\;\;\frac{1+4a\xi_{s}}{1+4b\xi_{s}}$$

The values of *R* are acquired by solving Equations (19) and (20) for the variable damping ratios (a and b) of the VDS, employing the modal response as input. The structure’s damping ratio can be estimated by solving the quadratic equation for the modal damping ratio, as presented in Equation (22). When the input comprises velocity and acceleration, the modal damping ratio can be determined through the solution of a linear equation.

### 2.4. Damping Ratio Estimation by Polychromatic Mode Input

Since the transfer function of the state-space mode with non-classical damping contains the polychromatic components, the response of the VDS can be expressed as a sum of the weights of the variance of each component in Equation (21). For the displacement and velocity components, the response can be given as

(23) $$\begin{array}{c} E\left[{u_{t}}^{2}|\eta =disp.,\eta =vel.\right]\\ \;\;\;\;\;\;=\frac{\beta \pi S_{o}}{8}\frac{1}{{\omega_{t}}^{7}}\frac{1+4\xi_{t}\xi_{s}}{\left(\xi_{t}+\xi_{s}\right)\xi_{t}\xi_{s}}+\frac{\alpha \pi S_{o}}{8}\frac{1}{{\omega_{t}}^{5}}\frac{1}{\left(\xi_{t}+\xi_{s}\right)\xi_{t}\xi_{s}}\\ \;\;\;\;\;\;=\frac{\pi S_{o}}{8{\omega_{t}}^{7}}\frac{\alpha {\omega_{t}}^{2}+\beta \left(1+4\xi_{t}\xi_{s}\right)}{\left(\xi_{t}+\xi_{s}\right)\xi_{t}\xi_{s}}\\ \alpha +\beta =1 \end{array}$$

Substituting the damping ratio of the VDS, *a* and *b*, to calculate the ratio *R*, the quadratic equation in terms of the modal damping ratio can be established to estimate the modal damping ratio of the structure as follows:

(24) $$A_{2}{\xi_{s}}^{2}+A_{1}\xi_{s}+A_{o}=0$$

$$\begin{array}{cc} A_{2}=4\beta (b\bar{R} & -\;a),\\ & A_{1}=\left(\alpha {\omega_{t}}^{2}+\beta +4\beta ab\right)\left(\bar{R}-1\right),\\ & A_{o}=\left(\alpha {\omega_{t}}^{2}+\beta \right)\left(a\bar{R}-b\right)\\ & \bar{R}=\frac{a}{b}R \end{array}$$

## 3. Numerical Simulation

To validate the presented technique, a simulation was conducted on a structure utilizing passive vibration control systems—specifically, the oil damper and TMD. The structure experienced a low-pass filtered white noise load. Simulated structural acceleration served as the measured response for identifying dynamic properties using the proposed method. A comparison between the outcomes obtained from the proposed method and the analytical solution was performed to evaluate the technique’s effectiveness.

### 3.1. Structure with an Oil Damper

**Figure 1:**
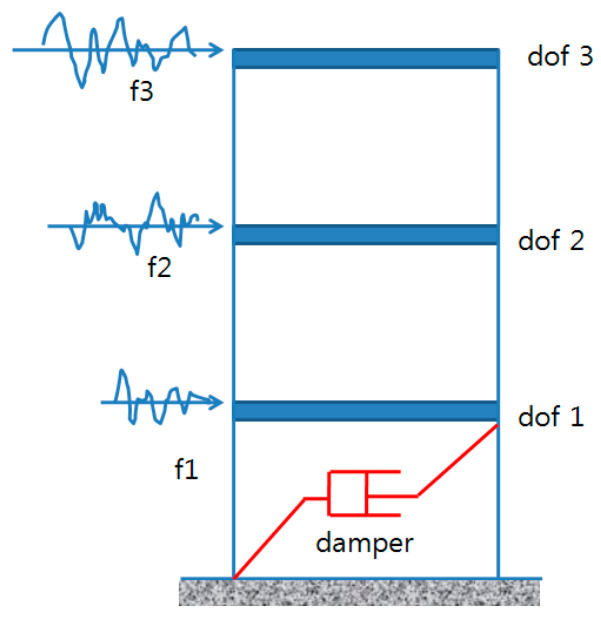
The reduced model with an oil damper.

Figure 1 depicts a three-degree-of-freedom analytical model featuring an oil damper in the first level, utilized to validate the proposed technique. The model simulates a 50-story high-rise building exposed to wind loading. The building model’s fundamental frequency is approximately 0.2 Hz, considering only its first three modes. Details about the 3-DOF model are provided in Table 1. The total non-classical damping comprises the combined effect of the 2% classical damping inherent to the structure and the additional damping introduced by the oil damper. Dynamic properties are assessed through eigenvalue analysis of the state-space system matrix A, derived from Equation (1) by converting from the MCK form. The analysis reveals that the natural frequency remains unaltered, while the damping ratio of the first mode increases from 2% to 10%. Notably, the second mode experiences a greater increase in damping ratio compared to the third mode. This discrepancy is attributed to the varying mode participation and the influence of the damper’s installation location at different levels.

**Figure 2:**
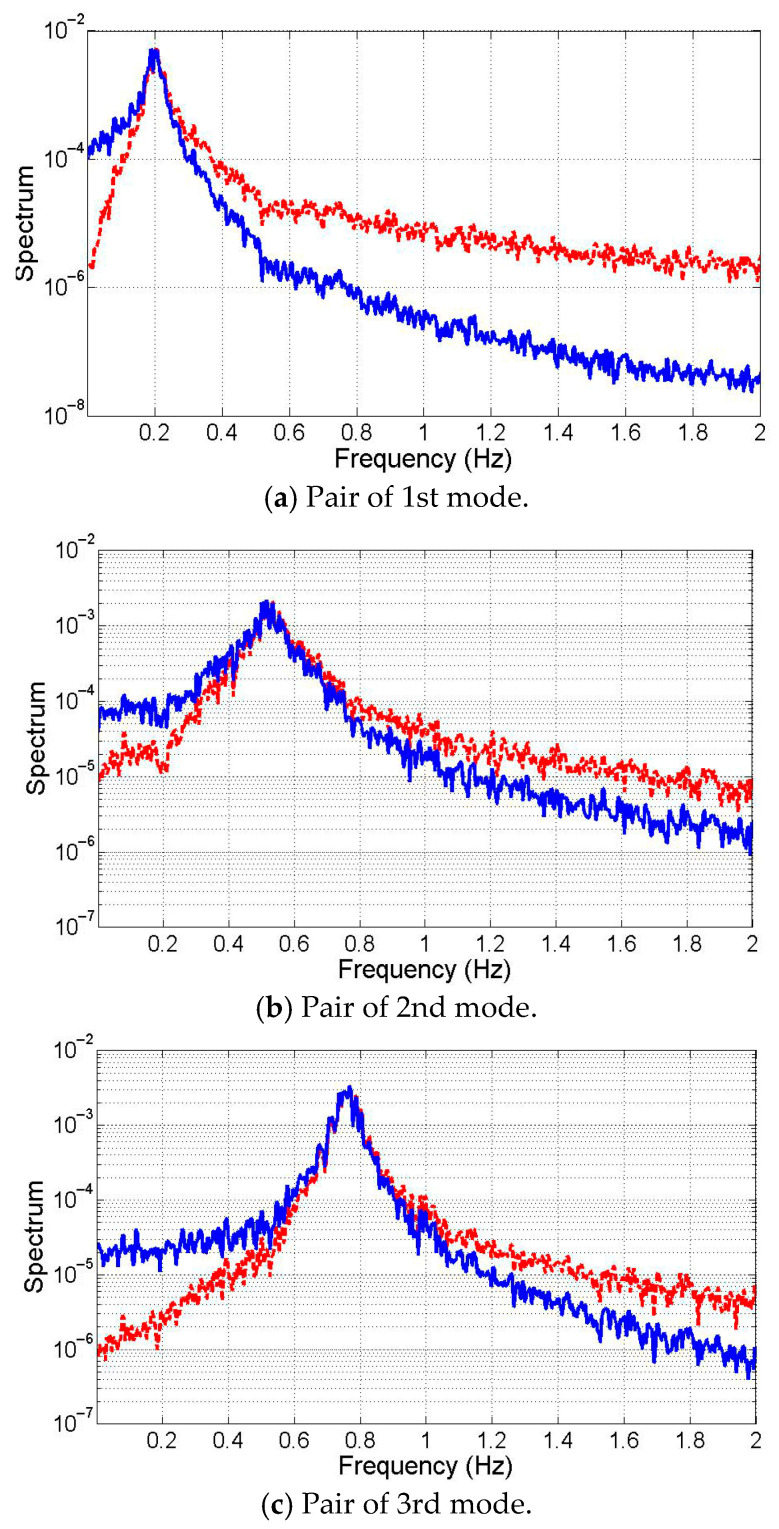
The separated mode in the state–space.

The model’s measured response was estimated by simulating acceleration at each degree of freedom under white noise loading. The sampling frequency is 10 Hz. Numerical integration of the simulated displacement and velocity established the state variable. The demixing matrix W was calculated using Equations (5) and (11) in the state-space to isolate each mode. Figure 2 displays averaged spectra of six modes: three conjugate pairs at each natural frequency. Spectra amplitude is log-scaled for frequency domain visualization. It is noted that two modes exist at a natural frequency. On either side, one mode’s low-frequency component dominates (blue curves), while another mode’s high-frequency component prevails (red curves).

Clearly, the state-space mode decomposition effectively separates three pairs of modes from the response data. Slight spectrum curve distortion near adjacent natural frequencies (0.5 Hz for Figure 2a and 0.2 Hz for Figure 2b, respectively) is observed. For instance, in Figure 2b, a sharp decline at 0.2 Hz (the first mode’s natural frequency in Figure 2a). However, amplitude change near other mode frequencies is negligible (e.g., 1/100 amplitude), preserving monochromaticity of separated modes.

The following transfer function is established in order to identify the polychromatic characteristics of the decomposed mode.

(25) $$T_{r}\left(s\right)=\frac{\alpha s}{s^{2}+2\xi_{1}\omega_{1}s+{\omega_{1}}^{2}}+\frac{\beta }{s^{2}+2\xi_{1}\omega_{1}s+{\omega_{1}}^{2}}$$

Equation (25) models the decomposed modal response as a linear combination of transfer functions corresponding to velocity and displacement inputs. The use of *α*s and *β* in the numerator reflects the fact that state-space modes inherently contain both velocity and displacement components, each contributing differently to the overall frequency response. While each term shares a common denominator representing the same resonant pole (i.e., identical natural frequency and damping), the numerators introduce different zero dynamics, giving rise to the polychromatic behavior observed in the modal spectrum.

By adjusting the weights *α* and *β*, the proposed transfer function flexibly captures the mixed spectral characteristics of the state-space-separated mode. This formulation allows us to quantify the influence of polychromatic components and thereby improve the accuracy of damping ratio estimation. The curve-fitting process minimizes the error between the empirical spectrum and the composite transfer function, leading to an optimal set of weights, as presented in Table 2.

The normalized positive weights (*α* and *β*) represent the transfer functions for velocity and displacement, similar to Equation (23). Through iterative adjustments of these weights, optimal *α* and *β* values were determined. This iterative process aimed to minimize the error between the transfer function and the modal spectrum using curve fitting. The resulting optimized weights for each mode are presented in Table 2.

**Figure 3:**
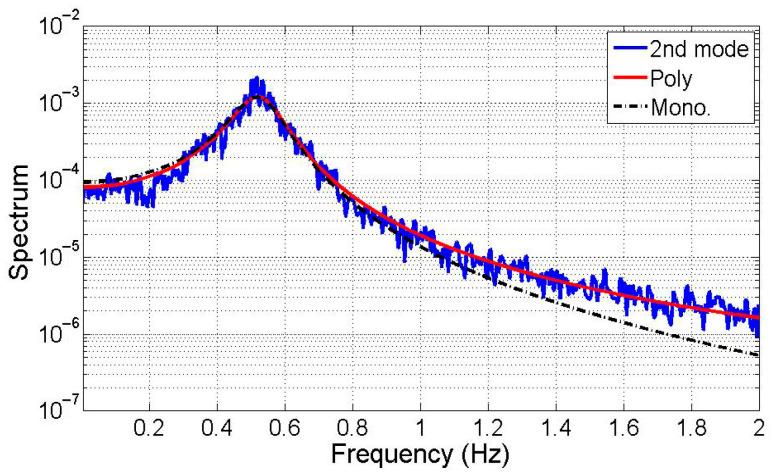
Comparison of the 2nd mode with the spectrum model.

Table 2 values reveal symmetrical *α* and *β* for each mode, where the low-frequency dominant spectrum’s *α* and *β* closely resemble the high-frequency dominant spectrum’s *β* and *α*. Notably, the second mode’s pronounced polychromatic properties stem from significant damping effects. In Figure 3, a comparison between the low-frequency dominant mode’s spectrum and the optimal transfer functions of the second mode highlights the substantial impact of polychromatic components, alongside the displacement monochromatic transfer function. The overlapping curves indicate similarity between the polychromatic and monochromatic transfer functions near the mode’s natural frequency. However, disparities among the spectra increase with rising frequency. This observation suggests representing the state-space mode with its inherent polychromatic component.

**Figure 4:**
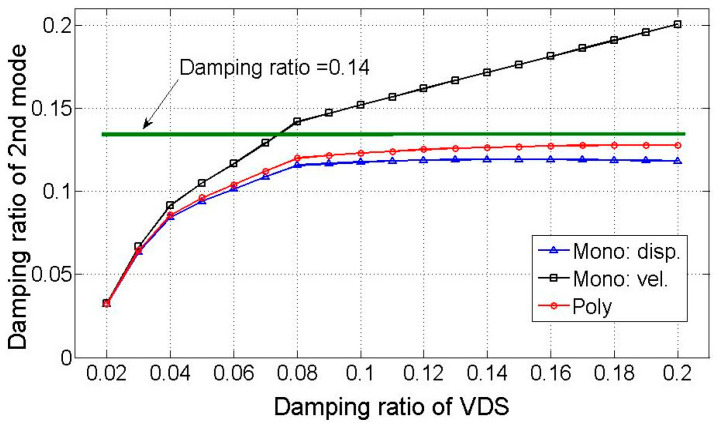
Estimation of damping ratio by VDS.

The modal damping was subsequently calculated using the estimated modal response as input to the VDS. Assuming the decomposed mode comprises a polychromatic component from displacement and velocity, Equation (22) estimates the modal damping. Figure 4 compares the resulting damping ratios of the low-frequency dominant mode with the estimated damping ratio based on the modal response incorporating the monochromatic component of displacement and velocity.

The VDS-derived estimated damping ratio ranges from 2% to 20%. Due to the greater weight of the displacement component compared to the velocity component in the low-frequency dominant second mode (Table 2), the damping ratio from the displacement monochromatic component closely aligns with the estimate from the polychromatic component. Although the estimated damping ratio from the polychromatic component is lower than the analytical value, its spectral response more closely matches the actual modal behavior. This suggests that estimating the damping ratio using the modal response established with the polychromatic component yields greater accuracy. For the first and third modes, where monochromatic components are prominently dominant as presented in Table 2, the VDS effectively estimates the damping ratio.

### 3.2. TMD–Structure System

This section presents a numerical analysis of a high-rise building equipped with a tuned mass damper (TMD) system designed to mitigate wind-induced vibrations. The TMD system introduces two distinct modes: a control mode for the structure and a mode for the TMD itself, collectively forming a non-classical damping system. Through state-space decomposition, we evaluate the damping ratio for each mode using a proposed technique.

Table 3 displays the selected mode for numerical simulation and TMD’s dynamic properties. The two-degree-of-freedom equation of motion, based on these properties, is transformed into a state-space equation. Eigenvalue analysis of this equation yields the natural frequency and damping ratio for both structure and TMD modes, presented in Table 3. To assess estimation accuracy, the relative errors between the in situ measurements and analytical values were calculated. The damping ratio exhibited relative errors of approximately 15.6% for the first mode and 14.0% for the second mode. In contrast, the natural frequency showed much smaller errors, with 0.75% and 0.19% for the first and second modes, respectively. These results demonstrate the method’s effectiveness in frequency estimation, while highlighting the sensitivity of damping estimation under experimental conditions. The second mode exhibits higher estimation accuracy than the first, which may seem counterintuitive. Although the first mode generally has a higher signal-to-noise ratio, in systems with TMDs, it is more strongly coupled with the damping device. This interaction introduces polychromatic effects and spectral distortion, complicating accurate parameter extraction. In contrast, the second mode is less affected by the TMD and retains a cleaner response, leading to more precise estimation.

**Figure 5:**
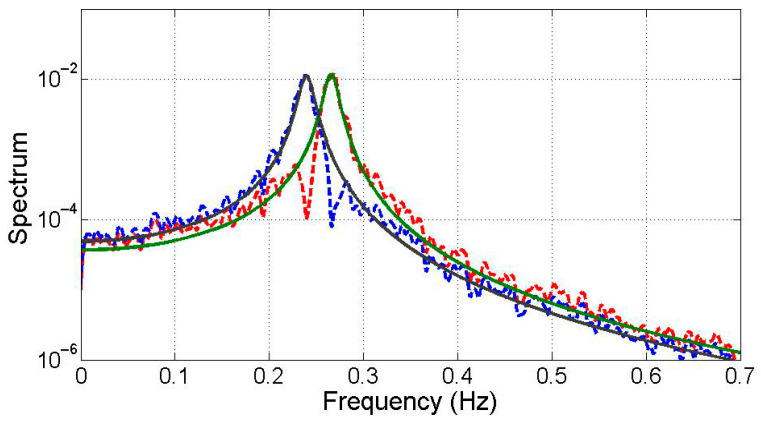
Decomposed modes.

**Figure 6:**
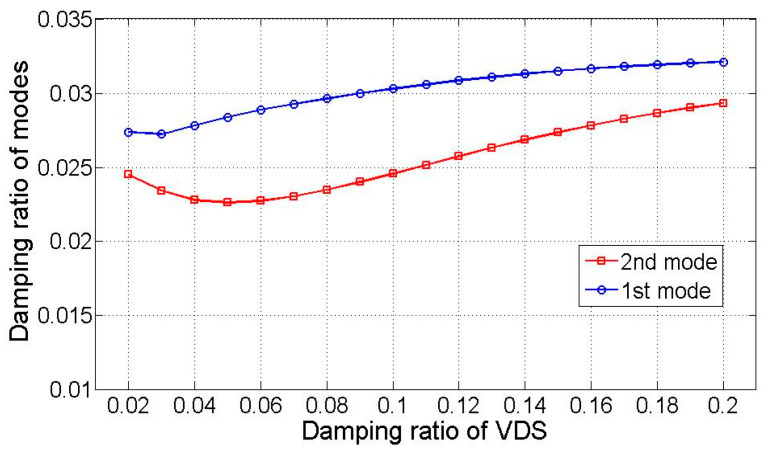
Estimated modal damping ratios as a function of the VDS damping ratio.

Integration of simulated acceleration responses of the TMD and structure derives state variables. These variables are then subjected to Equations (5) and (11), enabling the extraction of four modes using the demixing matrix W. Figure 5 illustrates the dominant low-frequency modes and compares them with optimized transfer functions primarily composed of displacement-based monochromatic components. The black solid line represents the analytical reference response, while the green solid line shows the optimized transfer function using only displacement components. The red dashed line corresponds to an estimated response including velocity or mixed inputs, and the blue dashed line represents the modal response extracted from heterogeneous signals via state-space separation. The latter two exhibit greater spectral variation due to polychromatic effects. Figure 6 showcases the input of monochromatic component modal responses into the VDS to assess dynamic properties. The “Estimated (numerical)” row in Table 3 provides averaged damping ratio and natural frequency estimates, which closely align with analytical solutions, except for a 10% discrepancy in the damping ratio.

### 3.3. Experimental Test

**Figure 7:**
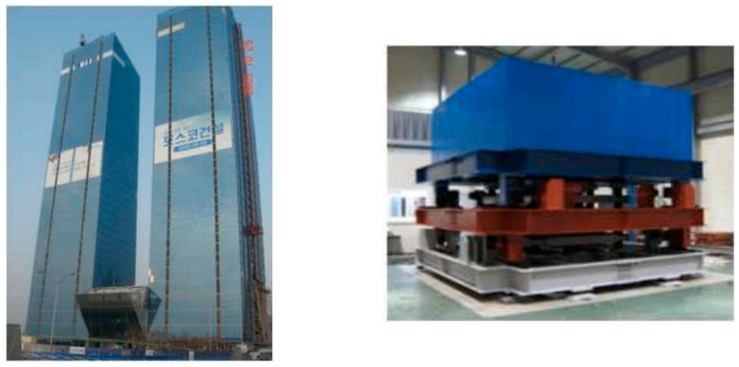
Building with TMD.

**Figure 8:**
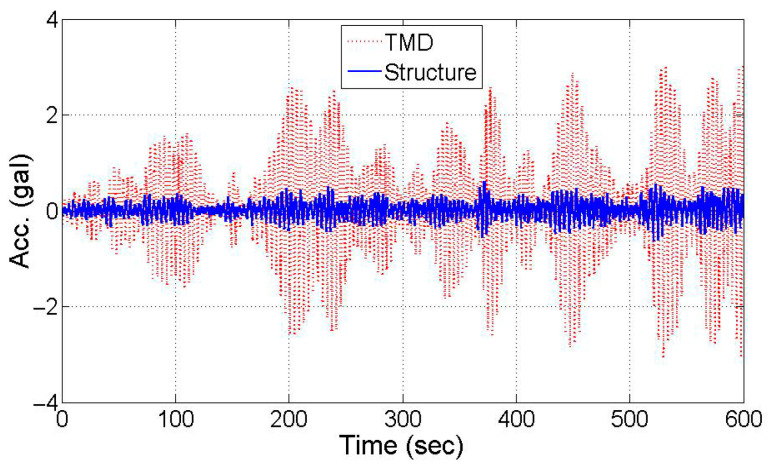
Time history of the experimental data.

**Figure 9:**
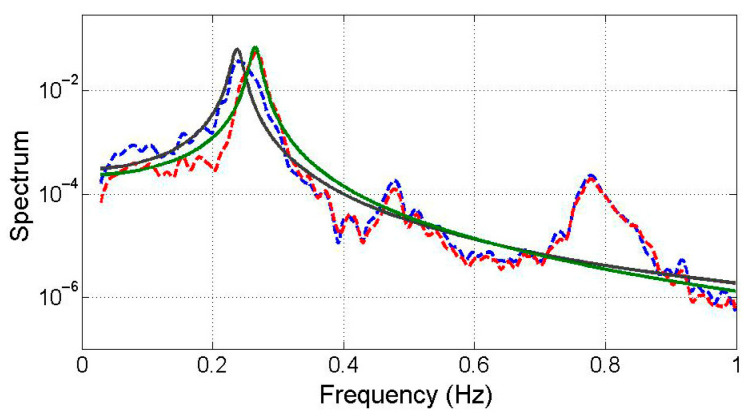
Decomposed modes from the experimental data.

**Figure 10:**
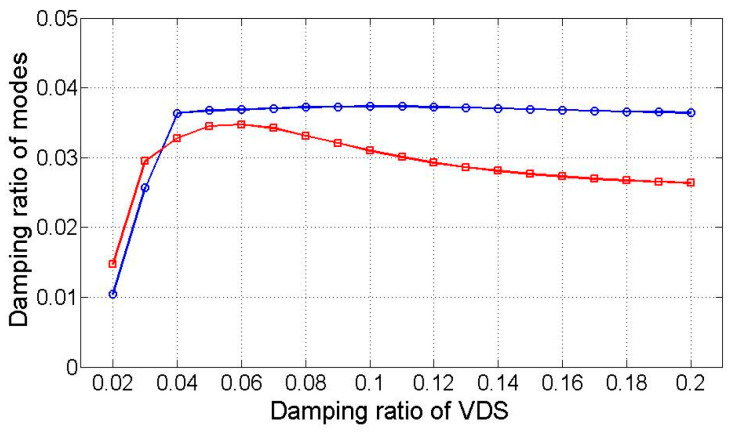
The damping ratio of the target structure.

Section 3.2 exemplifies the efficacy of the proposed dynamic property estimation method through numerical simulation for structure–TMD systems. Utilizing in situ data from the existing structure with TMD [17] shown in Figure 7, previously used for experimental validation, dynamic properties are identified. Figure 8 illustrates the time history of TMD acceleration and high-rise building motion under wind load. TMD peak acceleration during tower vibration control is 3 gal (3 cm/s^2^), sixfold that of the building. Integration of this acceleration yields state variables for mode decomposition (Figure 9). Figure 9 shows the modal responses extracted from in situ acceleration data of a high-rise building equipped with a TMD. The red and blue dashed lines represent modes separated from the measured acceleration signals using the proposed state-space decomposition method. The black and green solid lines correspond to optimized transfer functions, constructed to match the analytical modal characteristics using displacement-based monochromatic components. Two closely spaced TMD and structure modes are separated through this process, alongside peaks near 0.5 Hz and 0.8 Hz in the spectra. These correspond to the optimized transfer function components at adjacent modes. The damping ratios corresponding to the first (blue) and second (red) modes are presented in Figure 10, showing good agreement with analytical values. Predicted dynamic properties exhibit slight discrepancies, attributed to load and measurement conditions. The proposed method’s reliability for non-classically damped structures is demonstrated. Notably, for highly damped structures (Section 3.1), polychromatic mode characteristics emerge, contrasting monochromatic patterns in Section 3.2 and Section 3.3. This is due to correlation reduction caused by structural damping between 3% and 4% overshadowed by non-classical damping due to the TMD. Addressing this limitation through polychromatic mode analysis can enhance damping estimation accuracy.

Moreover, it is essential to highlight the significance of the proposed method’s versatility in accommodating various load and measurement conditions. The successful validation, encompassing both white noise input and real wind load scenarios in Section 3.2 and Section 3.3, underscores its robustness. Despite the differing duration of measurement (1 h and 10 min), the resulting damping estimates maintain an error below 10%, reaffirming the method’s reliability.

The presented research also sheds light on a noteworthy aspect: the interplay between damping characteristics and mode behavior. The transition from monochromatic to polychromatic mode expression, as observed in Section 3.1, Section 3.2 and Section 3.3, elucidates the impact of structural damping magnitude. For structures exhibiting substantial damping (Section 3.1), polychromatic features emerge due to diminished mode correlation resulting from low structural damping relative to non-classical damping. This limitation, however, can be mitigated by harnessing polychromatic properties within the state-space mode, offering an avenue for refining damping estimation precision.

The experiment using the existing building reveals that the proposed method showcases its reliability and adaptability in dynamic property estimation for structures characterized by non-classical damping. The comprehensive validation across different conditions, along with insights into mode behavior and damping, underscores the method’s potential to enhance the accuracy and applicability of dynamic property assessment in diverse engineering contexts.

## 4. Conclusions

This study introduces a novel framework for estimating damping ratios in non-classically damped systems by decomposing state-space modal responses and analyzing their frequency domain structures. Unlike conventional approaches, which assume modal responses to be inherently monochromatic, the proposed method acknowledges and quantifies polychromatic characteristics in the numerator of the modal transfer function. This innovation allows for a more precise and physically interpretable estimation of damping ratios, especially in structures equipped with oil dampers or tuned mass dampers.

A key finding of this study is the quantifiable relationship between the polychromatic ratio and the equivalent damping effect introduced by vibration control systems. As the equivalent damping ratio increases, the modal response exhibits a broader frequency spread in the numerator, indicating stronger polychromatic behavior. Conversely, in systems with low damping, the mode tends toward a monochromatic response, thus aligning more closely with classical assumptions.

These observations offer new insights into the spectral behavior of state-space modes under non-classical damping. In particular, the results suggest that the degree of polychromaticity can serve as a diagnostic indicator of damping device behavior. This relationship, which has not been explicitly addressed in the prior literature, provides a foundation for parameter tuning and health monitoring of damping systems.

Validation through both numerical simulations and experimental case studies confirms the robustness and reliability of the proposed method across different structural configurations and damping schemes. The case study involving oil dampers demonstrated the method’s sensitivity to variations in damping amplitude, while the structure equipped with tuned mass dampers highlighted the method’s applicability to frequency-tuned control systems.

The methodology also shows potential for broader applications in system identification, particularly in scenarios where classical assumptions are invalid due to the presence of complex damping mechanisms. Future research should focus on systematically quantifying the polychromatic ratio as a function of equivalent damping across a wider range of structural types and damper configurations. Additionally, it would be valuable to explore how external dynamic loads—such as vortex-induced wind loads, seismic inputs, or impulsive excitations—interact with the frequency composition of modal responses. Integrating the proposed framework with output-only identification techniques or machine learning-based monitoring systems may further enhance its utility in real-time structural health assessment.

By moving beyond the simplifying assumption of modal monochromaticity, this study offers a more realistic and detailed characterization of damping behavior in modern structural systems. It provides a foundation for more accurate analysis, improved control strategies, and the development of advanced monitoring techniques for structures employing non-classical damping.

## Figures and Tables

**Table 1:** Dynamic properties of 3-DOF model.

Item			Value																				
Mass matrix *M*			6.4896							0							0						
			0							5.4080							0						
			0							0							4.3264						
Stiffness matrix *K*			78.9568							−39.4784							0						
			−39.4784							78.9568							−39.4784						
			0							−39.4784							39.4784						
Classical damping matrix *C*			0.8620							−0.2464							−0.0508						
			−0.2464							0.7369							−0.2676						
			−0.0508							−0.2676							0.4629						
Non-classical damping matrix $C_{n}$			8		0		0		+		0.8620				−0.2464				−0.0508				
			0		0		0				−0.2464				0.7369				−0.2676				
			0		0		0				−0.0508				−0.2676				0.4629				
Mode			Mode 1							Mode 2							Mode 3						
Natural frequency (Hz)			0.2018							0.5282							0.7613						
Damping ratio (%)			9.56							14.17							4.45						

**Table 2:** The weight of the state-space mode model.

Mode	Weight (α and β)	
	Low-Frequency Dominant	High-Frequency Dominant
Mode 1	(0.0060 0.9940)	(0.9890 0.0110)
Mode 2	(0.1470 0.8530)	(0.8560 0.1440)
Mode 3	(0.0490 0.9510)	(0.9510 0.0490)

**Table 3:** Dynamic properties of structure and TMD.

Type			Item				Value				Unit			
Structure			Modal mass				13,453				ton			
			Natural frequency				0.256				Hz			
			Damping ratio				1.07				%			
TMD			Moving mass				160				ton			
			Mass ratio				0.0119				-			
			Natural frequency				0.249				Hz			
			Damping ratio				5.1				%			
System Matrix A			0			0			1.0000			0		
			0			0			0			1.0000		
			−2.6164			0.0291			−0.0363			0.0019		
			−2.4477			−2.4477			0.1596			−0.1596		
Eigenvalue							1st mode				2nd mode			
			Damping ratio				3.3467%				2.8453%			
			Natural frequency				0.2397 Hz				0.2659 Hz			
Estimated (numerical)			Damping ratio				3.0212%				2.5568%			
			Natural frequency				0.2389 Hz				0.2661 Hz			
Estimated (in situ)			Damping ratio				3.4923%				2.9155%			
			Natural frequency				0.2371 Hz				0.2666 Hz			

## Data Availability

The data that support the findings of this study are available from the corresponding author upon reasonable request.
